# The prognostic significance of survivin expression in patients with HNSCC: a systematic review and meta-analysis

**DOI:** 10.1186/s12885-021-08170-3

**Published:** 2021-04-17

**Authors:** Liu-qing Zhou, Yao Hu, Hong-jun Xiao

**Affiliations:** 1 Department of Otorhinolaryngology, Union Hospital, Ongji Medical College, Huazhong University of Science and Technology, Wuhan, 430022 China; 2 Department of Otorhinolaryngology, The Central Hospital of Wuhan, Huazhong University of Science and Technology, Wuhan, 430000 China

**Keywords:** Survivin, Head and neck squamous cell carcinoma, Prognosis, Meta-analysis

## Abstract

**Background:**

Survivin has been recently identified as a promising novel therapeutic target and prognostic marker in different types of cancer. Here we conducted a comprehensive meta-analysis to better clarify they the precise prognostic and diagnostic value of survivin in head and neck squamous cell carcinoma (HNSCC).

**Methods:**

Database of PubMed (Medline), Embase, and Web of Science were systematically searched for related published literature up to September 2020. Pooled hazards ratios (HR) and related 95% confidence intervals (CI) were used to estimate the association of survivin expression and survival outcomes in HNSCC patients.

**Results:**

Twenty eight studies with 4891 patients were finally included in this meta-analysis, the pooled analysis indicated that the survivin expression was significantly correlated with poorer overall survival (OS) (HR, 2.02; 95% CI, 1.65–2.47, *P* < 0.001), and poorer disease-free survival (DFS)/ disease-specific survival (DSS) (HR = 2.03, 95%CI: 1.64–2.52, *P* < 0.001; HR = 1.92, 95%CI: 1.41–2.60, *P* < 0.001, receptively). Similar results were observed in subgroup analysis stratified by different cancer types, such as laryngeal squamous cell carcinoma (LSCC) (HR = 1.35, 95%CI: 1.05–1.74, *P* < 0.001), oral squamous cell carcinomas (OSCC) (HR = 2.45, 95%CI: 1.89–3.17, *P* < 0.001), nasopharyngeal carcinoma (NPC) (HR = 2.53, 95%CI: 1.76–3.62, *P* < 0.001) and HNSCC (HR = 1.52, 95%CI: 1.25–1.86, *P* < 0.001). Furthermore, ethnicity-stratified analysis indicated that survivin was significantly associated with poorer OS among both Asian and Non- Asian HNSCC patients (HR = 2.16, 95%CI: 1.76–2.66; HR = 1.56, 95%CI: 1.33–1.82, respectively).

**Conclusions:**

Our results suggested that survivin is predictors of worse prognosis in HNSCC patients. Hence, survivin is a potential therapeutic target for HNSCC.

**Supplementary Information:**

The online version contains supplementary material available at 10.1186/s12885-021-08170-3.

## Introduction

Head and neck squamous cell carcinoma (HNSCC) is ranking as the sixth most prevalent cancer worldwide [1], they develop from the squamous mucosa of the upper aerodigestive tract, including nasal cavity, nasopharynx, larynx, hypopharynx, oropharynx and so on. Squamous cell carcinoma (SCC) accounts for up to 90% of malignant tumor in the head and neck region. The group of malignancies have similar pathogenesis, staging system, therapeutic strategy, and prognosis despite they arise from different sites of head and neck region, hence, it is rational to classify them into one category, HNSCC [2]. There are several risk factors associated with HNSCC, such as environmental exposures, tobacco use, alcohol consumption and so on [3]. Although the diagnosis and multimodality treatments improved quickly, the 5-year survival rate still remains very low due to the complex anatomy of head and neck region [4]. Therefore, it is necessary to identify more reliable new prognostic biomarkers and therapeutic targets.

Survivin, an important member of the ‘inhibitor of apoptosis’ (IAP) family, is encoded by baculoviral inverted repeats (BIR) C5 gene [5]. The expression is minimal in normal tissues, however, strong survivin expression is largely observed in numerous cancers [6]. Survivin is an anti-apoptotic factor and regulate intrinsic and extrinsic apoptotic pathways by interacting with many factors. It also plays key roles in cellular stress response by interfering with autophagy. Different proteins of the autophagic machinery, such as Beclin 1, chemokine ligand 2 and light chain 3 interact with survivin [7, 8]. In addition, survivin expression is associate with the process of angiogenesis, it overcomes G2/M phase of the cell cycle and causes mitotic progression in most adults’ cancers [9].

Although numerous researches have revealed the association between survivin and the prognosis of HNSCC, the results still remain controversial due to the variance in the sample size, study design, test approach and cut-off value. Therefore, it is essential to clarify the diagnostic and prognostic value of survivin in HNSCC based on the findings from the independently small sample size publications. Here, we searched PubMed (Medline), Embase, and Web of Science databases for relevant publications and undertaken a comprehensive meta-analysis to systematically assess the diagnostic and prognostic role of survivin in HNSCC.

## Methods

### Search strategy

We searched for articles published between 2000 and 2020. Electronic searches for relevant retrieve studies were performed throughout databases, including PubMed (Medline), Web of Science and EMBASE databases in accordance with Dickersin et al. in September 2020 [10]. The retrieval strategy included: (survivin) and (prognosis OR outcome OR mortality OR survival OR progression OR recurrence) and (head and neck or laryngeal or tonsil or oropharyngeal or oral or oropharynx or nasopharyngeal) and (squamous cell cancer or carcinoma). Furthermore, the reference lists of retrieved articles for additional articles were also manually searched. If several studies reported the same patient populations, we enrolled the most complete one to avoid duplication.

### Selection criteria

This meta-analysis was limited to studies about the association between HNSCC and survivin. The inclusion criteria of the meta-analysis were as follows: (1) all patients should be diagnosed with HNSCC; (2) survivin was evaluated in both samples of the HNSCC and normal controls; (3) studies revealed the association between survivin and survival of HNSCC; (4) sufficient statistical analysis was required, including hazard ratios (HR) and their related 95% confidence interval (95% CI)) for survival outcomes, if not we could calculate them by *p* values and Kaplan–Meier curves [11] (5) the language of publications was limited to English. The exclusion criteria were: (1) studies without sufficient data for meta-analysis; (2) abstracts, case reports, reviews, letters, expert opinions, etc.; (3) studies about cell lines, in vivo/vitro studies, and human xenografts. If the same cohort was reported by several studies, the most recent one was included in our study.

### Data extraction

We inspected the duplicates, and removed the repeated papers first. Then, we perused the titles and abstracts of the papers carefully. At last full articles were selected to include the appropriate studies. Two researchers independently evaluated the literature against the inclusion and exclusion criteria (LQ Zhou and Y Hu). Any discrepancy in assessments was resolved by consulting an adjudicating with a third researcher (HJ Xiao). The researchers of the studies were contacted by e-mail to request data or additional information for meta-analytic calculations. The eligible studies for this meta-analysis were reviewed by two reviewers (LQ Zhou and Y Hu) independently. The Newcastle–Ottawa Quality Assessment Scale (NOS) was included to assess the methodological qualities of each publication, a star system (range from 0 to 9) was adopted to evaluate a including publications in three domains, comparability of study groups (2 stars), selection of participants (4 stars) and the outcome measurement (3 stars). A study with NOS ≥ 6 was seemed as a high-quality study [12, 13]. Reporting recommendations for tumor marker prognostic studies (REMARK), which were developed to address widespread deficiencies in the reporting of such studies, was also applied to evaluate study quality in the present meta-analysis [14]. The REMARK checklist consists of 20 items to report for published tumor marker prognostic studies. They provide a comprehensive overview to educate on good reporting and also provide a valuable reference for the many issues to consider when designing, conducting, and analyzing tumor marker studies and, with minimal adjustment, in prognostic studies in medicine in general.

### Statistical analysis

The HR and the related 95% CI of survival outcomes were obtained directly from the primary publications or estimated by *p* values and other published data following Parmer’s methods [15]. Statistical heterogeneity among the studies was evaluated using the χ^2^-based Q test and the I^2^ statistics [16]. The fixed-effects model was employed for analysis without obvious statistical heterogeneity between studies (*P* > 0.10, I^2^ < 50%). Otherwise, the random-effects model was applied. Moreover, we performed subgroup analysis to explore the potential source of heterogeneity. Sensitivity analysis was conducted to investigate the influence of each individual study on the overall pooled results. We used the Begg’s and Egger’s tests to assess the potential publication bias. We conducted all statistical analyses by STATA statistical software version 15.0 (StataCorp Lp).

## Results

### Selection and characteristics of included studies

**Fig. 1:**
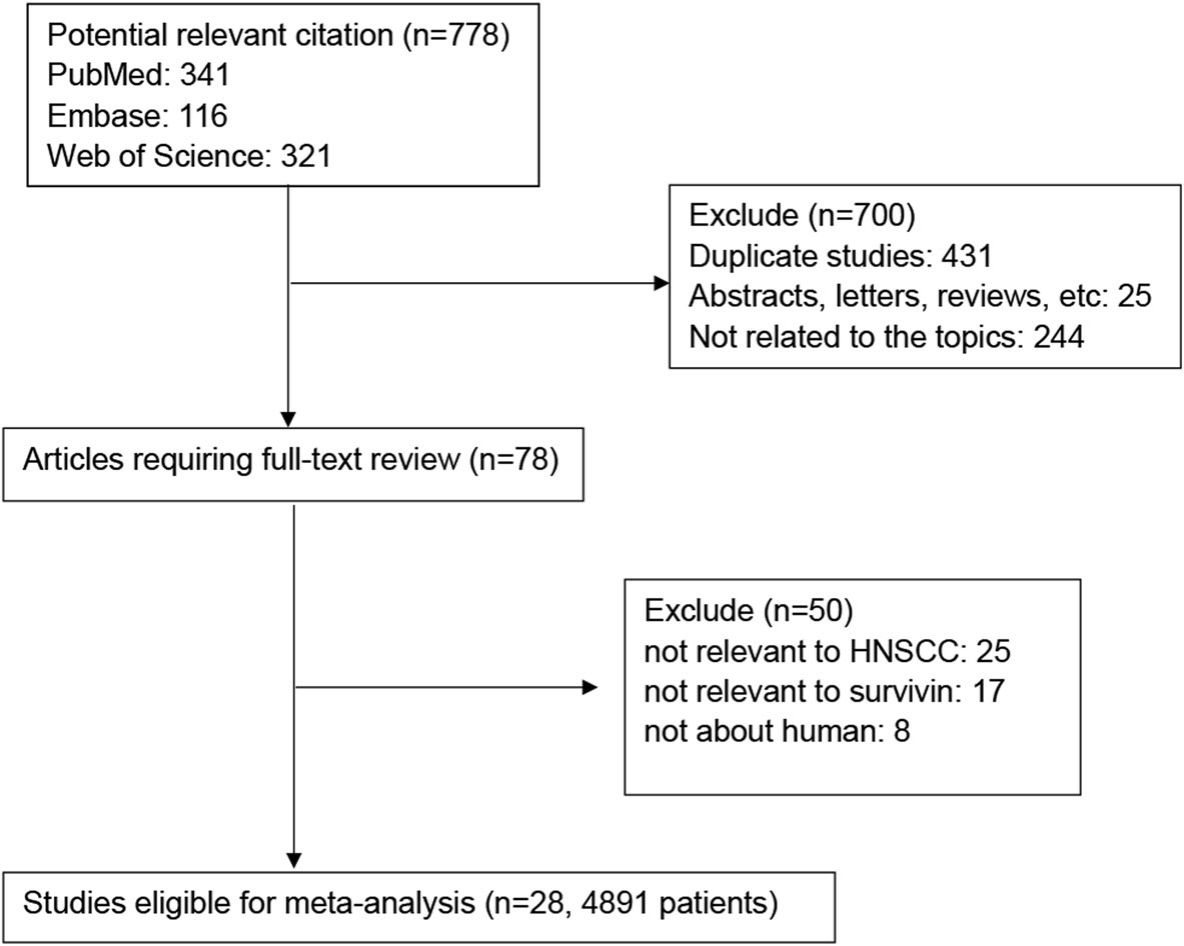
Flow diagram of the selection of relevant studies included in the meta-analysis

A total of 778 potential records were initially identified by searching the electronic databases (Fig. 1). Following exclusion of the duplicates (*n* = 431), reviews, abstracts and letters (*n* = 25) and the studies of irrelevant topics (*n* = 244), 78 studies were remained for further assessment by reading their full-text articles. A total of 50 studies did not provide specific data regarding HNSCC or survivin and therefore were excluded. Finally, a total 4891 HNSCC patients in 28 studies with publication years ranging from 2002 to 2019 were enrolled in the present meta-analysis.

The characteristics of the eligible studies were summarized in Table 1. The participants were from China, Tunisia, Turkey, Germany, Italy, Australia, Canada, South Korea, Sweden and Slovenia. Immunohistochemistry (IHC) and/or reverse-transcription polymerase chain reaction (RT-PCR) were used to analysis the survivin protein and/or mRNA expression in the including studies. The characters of antibodies in different studies are summarized in Table S1. They have different clones, dilutions, sources and positive signals. Different PCR instruments were also used in different studies. Our meta-analysis including eight studies for laryngeal squamous cell carcinoma (LSCC), ten studies for oral squamous cell carcinomas (OSCC), five studies for head and neck squamous cell carcinoma (HNSCC) and five studies for nasopharyngeal carcinoma (NPC). Fifteen publications included > 100 patients and 13 publications enrolled < 100 patients, twenty one studies including a total of 3036 patients reported overall survival (OS), nine studies including a total of 925 patients reported disease-free survival (DFS) and three studies including a total of 1485 patients reported disease-specific survival (DSS). The HR and 95% CI were directly reported in fifteen studies and were estimated in thirteen studies in the original literature. All of the publications’ NOS scores were above 6 and the REMARK scores were between 11 and 15.

**Table 1:** Characteristics of the studies examined in the meta-analysis

Author	Year	Country	Ethnicity	Cancer type	Sample size	Age	Follow-up (month)	Survival analysis	Method	Cut-off value	HR	NOS/REMARK score
Elhadj [17]	2019	Tunisia	Non-Asian	LSCC	70	63 (45–88)	NR	OS, DFS	IHC	5%	Reported	7/12
Erpolat [18]	2012	Turkey	Non-Asian	HNSCC	58	56.7 (35–80)	56.5 (38.7–112.5)	OS	IHC	5%	Reported	7/11
Fiedler [19]	2018	Germany	Non-Asian	HNSCC	139	60.5 (43.4–83.6)	17.4 (0–120.9)	OS,DSS	IHC	median value	Reported	6/14
Jin [20]	2019	China	Asian	NPC	164	45.0 (24–70)	49.2 (9–60)	OS,DMFS, LRFS,DFS	IHC, RT-PCR	0%	Reported	6/13
Li [21]	2008	China	Asian	NPC	280	46 (14–86)	NR	OS	IHC	5%	Reported	7/11
Li [22]	2011	China	Asian	LSCC	86	51 (37–84)	NR	OS	IHC	0%	Reported	7/12
Lin [23]	2005	China	Asian	OSCC	96	NR	NR	OS	IHC	25%	Reported	6/12
Muzio [24]	2005	Italy	Non-Asian	OSCC	78	66.5 (18–87)	72	DSS	IHC	75%	Reported	7/12
Preuss [25]	2008	Australia	Non-Asian	OSCC	106	57 (34–82)	20.3 (0.33–79.8)	DFS	IHC	5%	Reported	7/13
Tastekin [26]	2017	Turkey	Non-Asian	OSCC	46	59.48 (31–91)	NR	OS	IHC	13.00%	Reported	6/11
Wang [27]	2011	China	Asian	NPC	1268	46 (15–90)	69 (1–20)	DSS	IHC	median value	Reported	7/13
Xiang [28]	2006	China	Asian	NPC	80	NR	60	OS, DFS	IHC	25%	Reported	7/13
Yip [29]	2006	Canada	Non-Asian	NPC	198	NR	136.8	OS	IHC	5%	Reported	8/15
Zhao [30]	2008	China	Asian	LSCC	146	54.6 (42–76)	41.4 (36–72)	DFS	IHC	5%	Reported	7/14
Dong [31]	2002	Japan	Asian	LSCC	102	63.49 (38–89)	NR	OS,DFS	IHC	5%	Reported	7/13
Kim [32]	2005	South Korea	Asian	OSCC	113	58 (18–78)	NR	OS	RT-PCR	50%	Estimated	6/12
Zhang [33]	2013	China	Asian	OSCC	110	58 (37–78)	> 60	OS	IHC	median value	Estimated	7/13
Farnebo [34]	2013	Sweden	Non-Asian	HNSCC	40	68	30	OS	IHC	0%	Estimated	6/11
Freier [35]	2007	Germany	Non-Asian	OSCC	296	60 (16–92)	34 (0–147)	OS	IHC	10%	Estimated	7/13
Hansson [36]	2017	Sweden	Non-Asian	LSCC	149	NR	67 (9–163)	DFS	IHC	10%	Estimated	7/11
Munscher [37]	2019	Germany	Non-Asian	HNSCC	452	NR	41.3 (1–306)	OS,RFS	IHC	50%	Estimated	7/11
Pickhard [38]	2014	Germany	Non-Asian	HNSCC	180	53 (35–72)	60–162	OS	IHC	10%	Estimated	6/11
Su [39]	2010	China	Asian	OSCC	78	NR	NR	OS	IHC,RT-PCR	median value	Estimated	6/11
Troiano [40]	2018	Italy	Non-Asian	OSCC	342	NR	NR	OS	IHC	60%	Estimated	7/11
Pizem [41]	2004	Slovenia	Non-Asian	LSCC	68	59.2 (37–78)	NR	OS	IHC	50%	Estimated	6/12
Marioni [42]	2013	Italy	Non-Asian	LSCC	33	NR	43	DFS	IHC	10.00%	Estimated	6/11
Marioni [43]	2017	Italy	Non-Asian	LSCC	75	63.6	67.3	DFS	IHC	6%	Estimated	7/13
Kim [44]	2010	South Korea	Asian	OSCC	38	58.5 (40–75)	NR	OS	IHC	20%	Estimated	6/11

### Association between survivin and survival outcomes of HNSCC patients

**Fig. 2:**
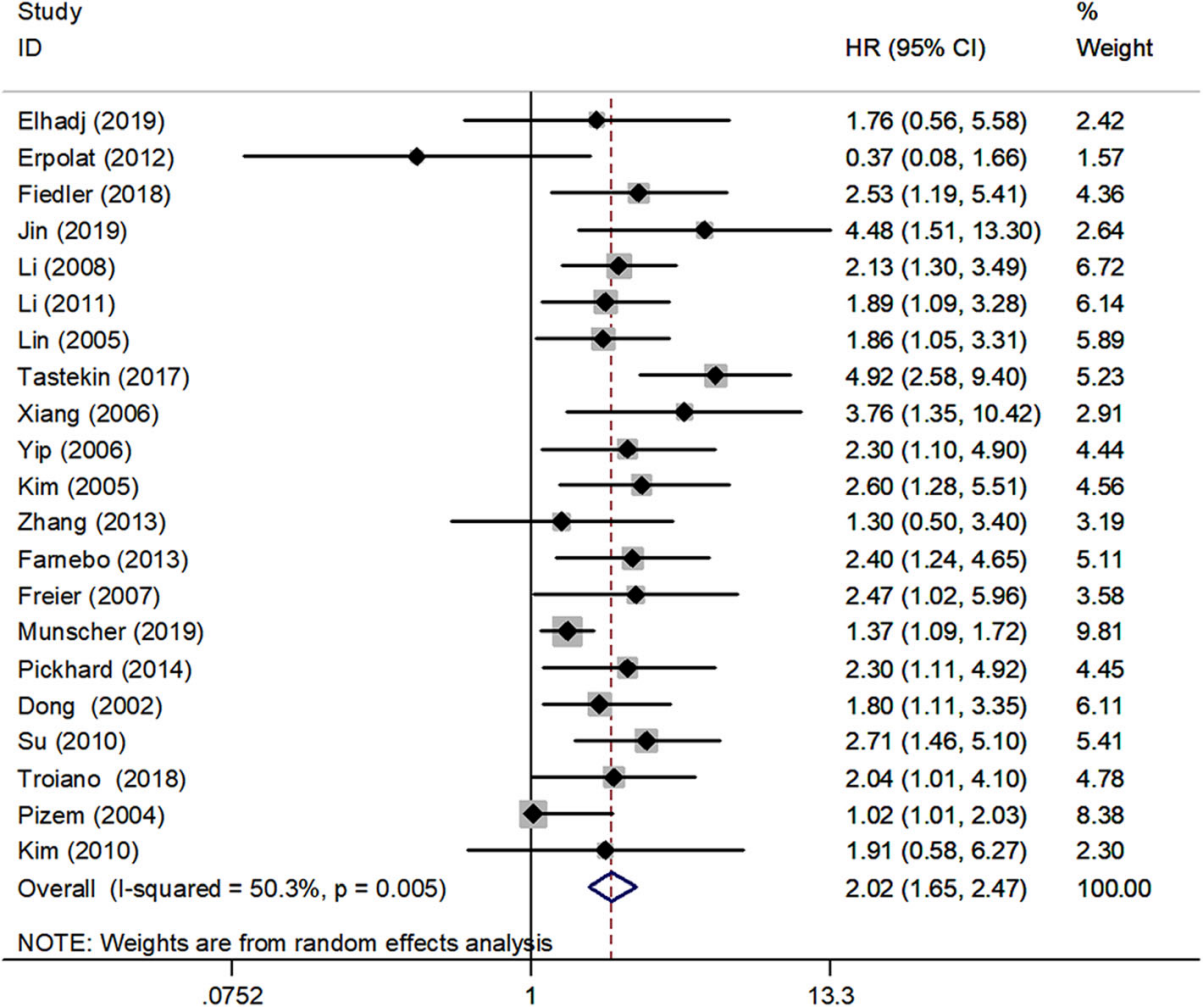
Forest plot indicating the association between survivin expression and OS in HNSCC

**Fig. 3:**
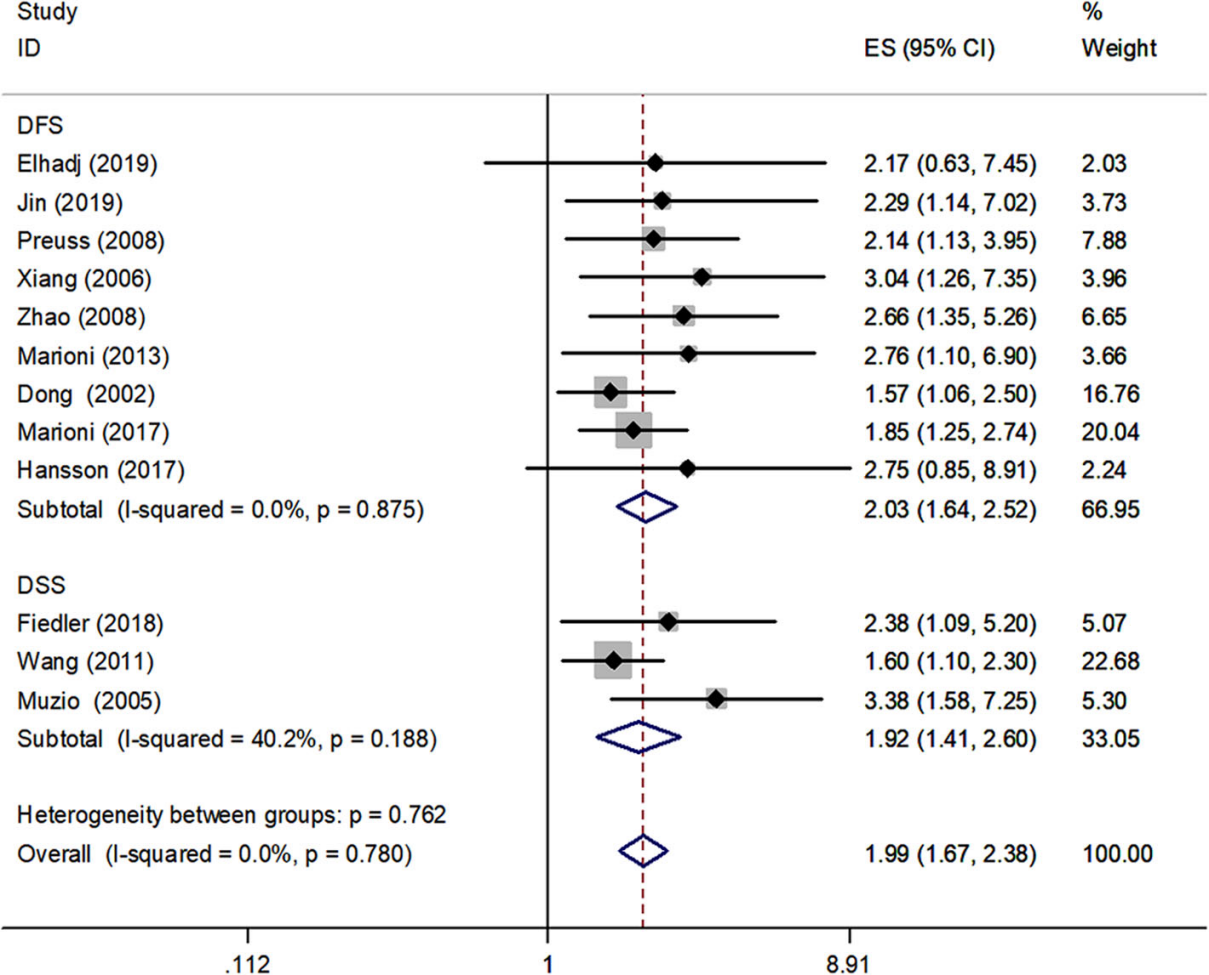
Forest plot examining the association between survivin expression and DFS/DSS in HNSCC

A total of twenty one studies in the present analysis examined the association between survivin and the OS of in HNSCC patients. The heterogeneity among the publications in our study was significant for Q test (*P* < 0.1). Hence, the random-effects model was adopted and subgroup analysis was used to seek for the potential causes of heterogeneity. The results of these studies indicated expression of survivin were associated with poorer OS (HR, 2.02; 95% CI, 1.65–2.47, *P* < 0.001). Medium heterogeneity was noted (I^2^ = 50.3%, P_heterogeneity_ = 0.005) (Fig. 2). Nine studies examined the association between survivin and the DFS and three studies examined the association between survivin and the DSS in HNSCC patients. Figure 3 summarized HR for DFS (HR = 2.03, 95%CI: 1.64–2.52) and DSS (HR = 1.92, 95%CI: 1.41–2.60), and there was no significant heterogeneity noted between survivin expression and DFS (I^2^ = 0.0%, P_heterogeneity_ = 0.875), low heterogeneity was noted between survivin expression and DSS (I^2^ = 40.2%, P_heterogeneity_ = 0.188).

### Subgroup analysis for OS was also performed and was stratified according to different cancer types or geographic populations

**Fig. 4:**
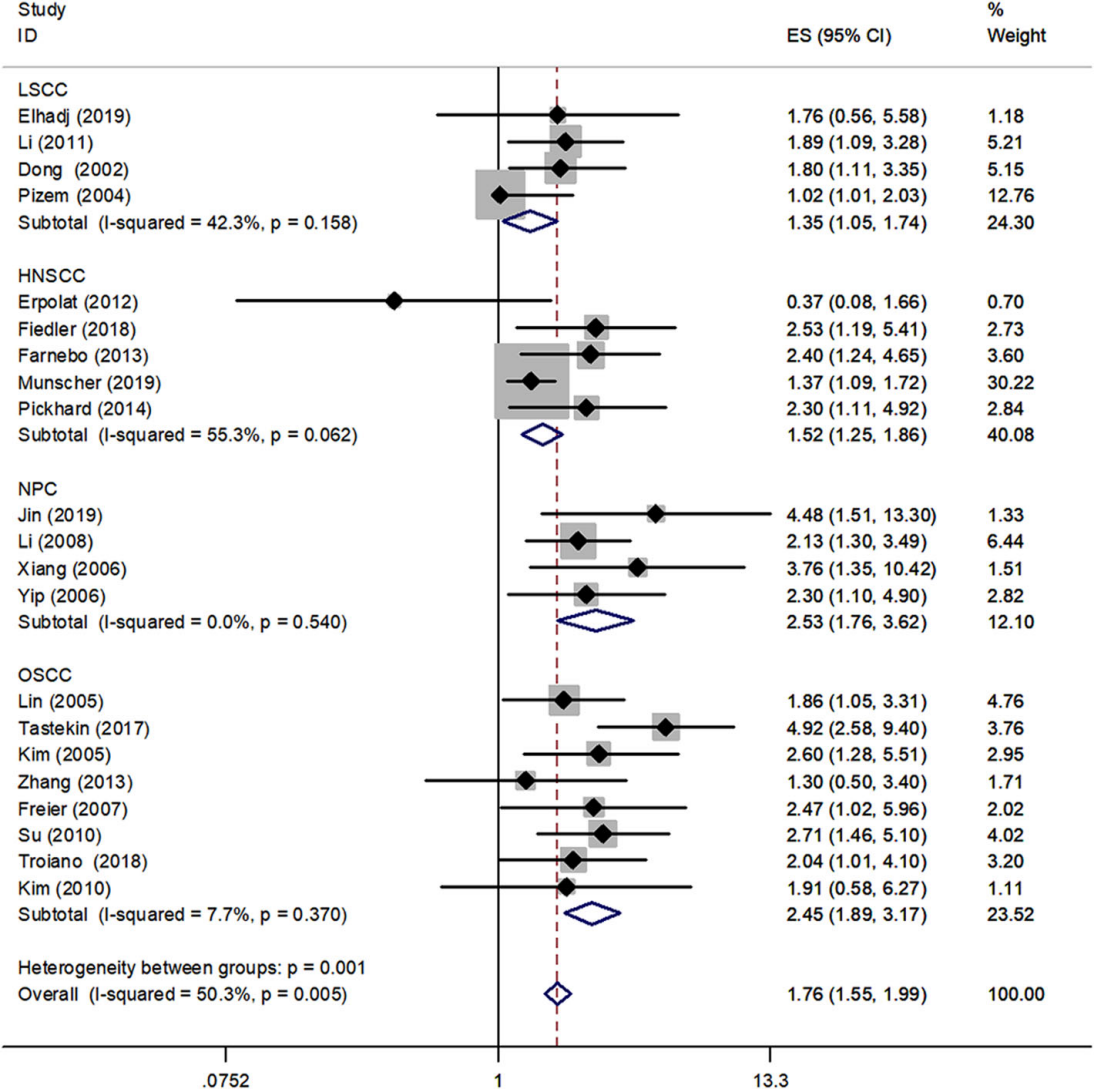
Forest plot of OS in association with survivin in different types of HNSCC

Cancer-stratified analysis showed that the summarized HR for LSCC was 1.35 (95% CI, 1.05–1.74, *P* < 0.001) with low heterogeneity (I^2^ = 42.3%, P_heterogeneity_ = 0.158); for HNSCC was 1.52 (95% CI, 1.25–1.86, *P* < 0.001) with medium heterogeneity (I^2^ = 55.3%, P_heterogeneity_ = 0.062); for NPC was 2.53 (95% CI, 1.76–3.62, *P* < 0.001) with no significant heterogeneity (I^2^ = 0.0%, P_heterogeneity_ = 0.540) and for OSCC was 2.45 (95% CI, 1.89–3.17, *P* < 0.001) with no significant heterogeneity (I^2^ = 7.7%, P_heterogeneity_ = 0.370) (Fig. 4).

**Fig. 5:**
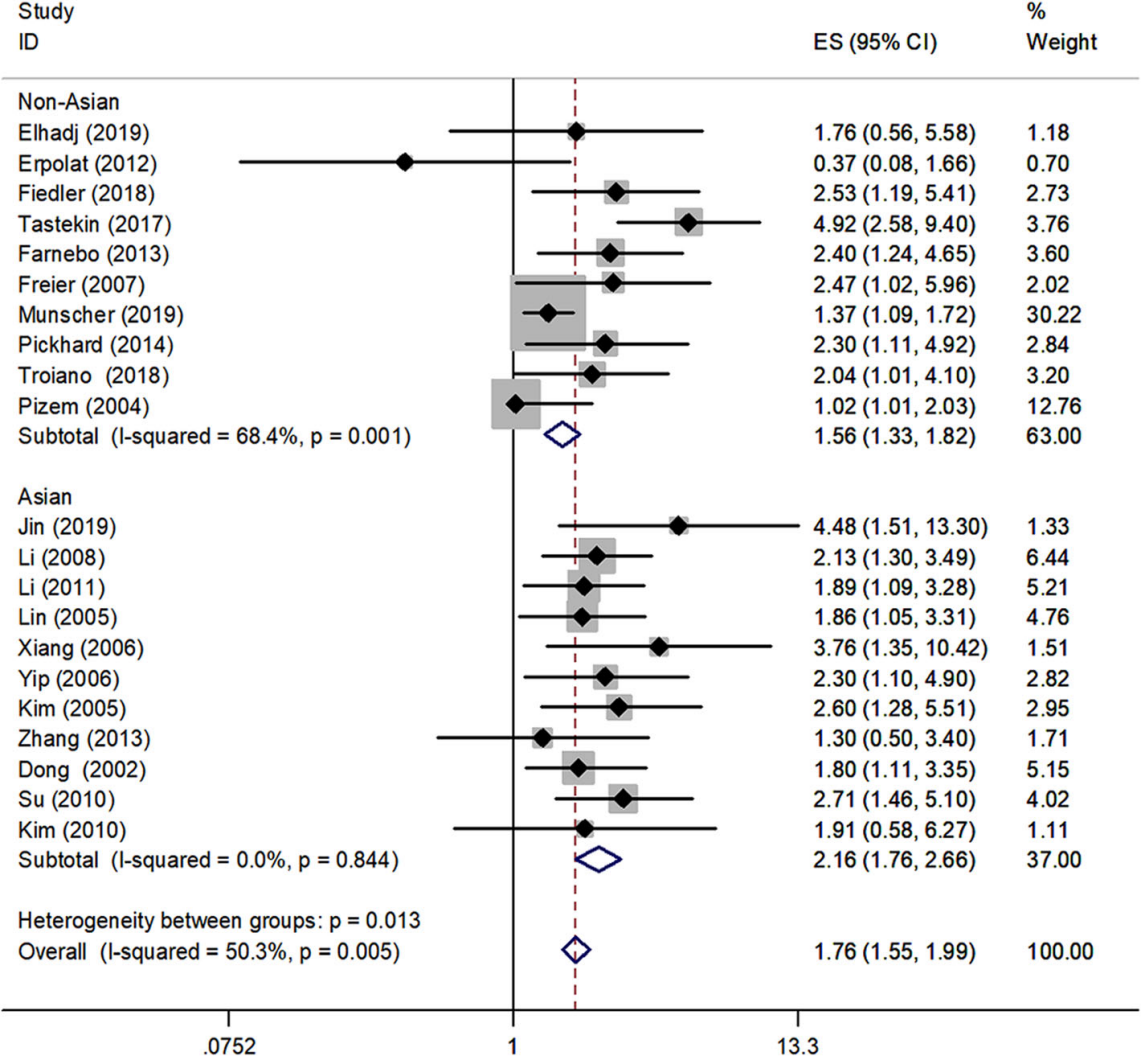
Forest plot of OS in association with survivin in different types of geographic populations

Ethnicity-stratified analysis demonstrated that the summarized HR for Non-Asian HNSCC patients was 1.56 (95% CI, 1.33–1.82, *P* < 0.001) with medium heterogeneity (I^2^ = 68.4%, P_heterogeneity_ = 0.001); for Asian patients was 2.16 (95% CI, 1.76–2.66, *P* < 0.001) with no heterogeneity (I^2^ = 0.0%, P_heterogeneity_ = 0.844) (Fig. 5).

**Fig. 6:**
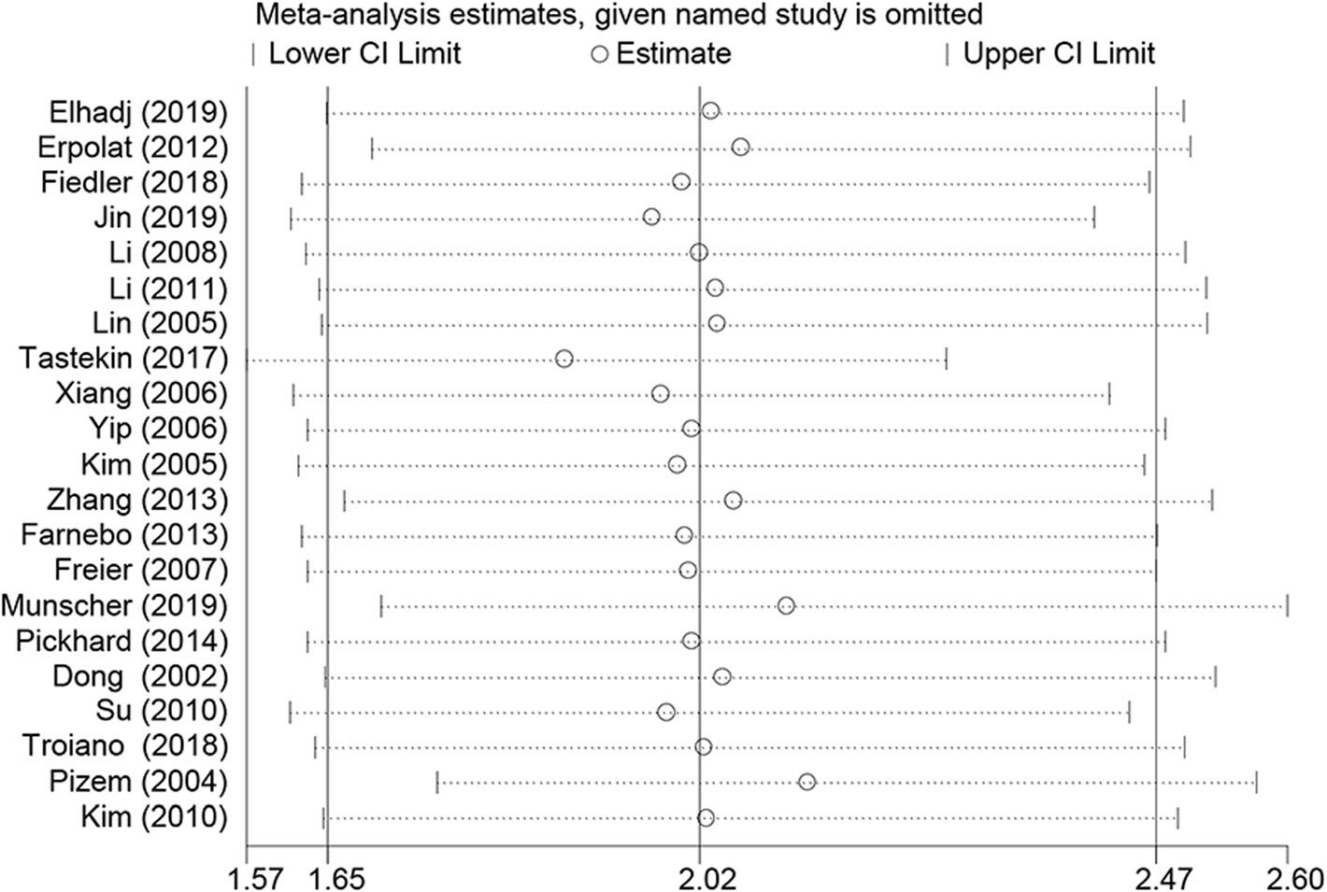
The sensitivity analyses were conducted to evaluate the effects of each single study on the overall effect

*Sensitivity analysis.* The sensitivity analysis was applied to detect the effects of each single study on the overall effect. The analysis did not detect a study that could alter significantly the combined results (Fig. 6). The results of the sensitivity analysis indicated that the pooled effect size of the meta-analysis results was stable and reliable.

### Publication bias

**Fig. 7:**
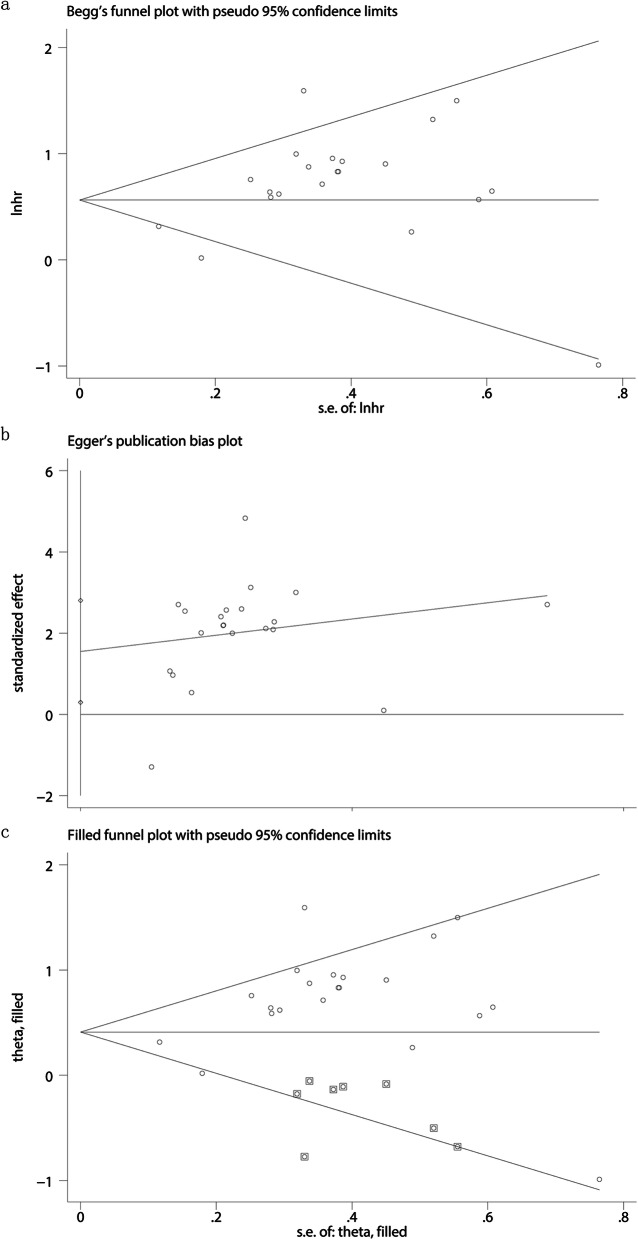
Publication bias and trim and fill analysis of the enrolled analysis. **a** The Begg’s funnel plots; **b** The Egger’s test. **c** Trim and fill analysis

The publication bias was assessed by the Begg’s funnel plots and the Egger’s test in the present study. The results indicated the publication bias existed among the studies (*p* = 0.018). Therefore, “trim and fill” analysis was further utilized, and the pooled HR of 1.569 (95% CI, 1.276–1.930) remained statistically significant (Fig. 7), therefore, the results of the present studies were robust in spite of the significant publication bias.

## Discussion

Numerous studies have focused on the identification of new prognostic biomarkers that can be used for cancer monitoring and detection. An association between survivin expression and survival outcomes has been shown in HNSCC patients. The study by Fiedler et al. found that high survivin expression was significantly correlated to unfavorable DSS in HNSCC patients [19]. Kim et al. demonstrated that survivin overexpression had a significant negative effect on survival outcomes of OSCC patients [44]. However, contradictory results were reported by Hansson et al. that patients with strong nuclear survivin expression had better 5-year DFS compared with those with weak nuclear survivin expression [36]. Furthermore, Kim et al. found that Asian patients diagnosed with non-nasopharyngeal head and neck cancer tended to exhibit better OS than non-Asian patients [45]. The present study is the first meta-analysis including 28 published studies with 4891 patients to provide useful information for clinical decision-making in HNSCC. Survivin was significantly associated with poorer OS in HNSCC patients, with HR values of 2.02, similar results were found in subgroup analysis stratified by cancer type, such as LSCC, OSCC, NPC, etc. Significant correlation between survivin and shorter DFS/ DSS (HR 2.03/ HR 1.92, respectively) was also observed. Moreover, ethnicity-stratified analysis showed that survivin was significantly associated with poorer OS among both Asian and Non- Asian HNSCC patients (HR 2.16/ HR 1.56, respectively). These findings confirmed that survivin could be widely applied as diagnostic markers and therapeutic targets in HNSCC patients.

The prognostic value of survivin was investigated in HNSCC and gathering evidences suggested that survivin was an independent prognostic marker in HNSCC [46, 47]. Epigenetic modifications play roles in HNSCC by regulating survivin expression [48]. The hypomethylation of BIRC5 is an important step in OSCC tumorigenesis due to its GC-rich region [49]. p53 also participates in the survivin upregulation in OSCCs, positive correlation was found between survivin expression and p53 in both HNSCC and premalignant lesions by Khan et al. [50]. The survivin gene locus encodes for multiple alternative splice variants with several different functions and heterodimerization possibilities [51]. Twenty three HNSCC cell lines at different differentiation levels showed higher survivin expressed levels compared to a human cell line of epidermal keratinocyte [52]. Targeted therapies have produced striking benefits for patients with cancers. Survivin is preferable targets of therapeutic modalities, namely antisense nucleotides, small-molecule inhibitors, antitumor immunotherapy and RNA interference [53, 54]. According to our results, the survivin inhibitors could be the therapeutics against the HNSCC. The expression of survivin in HNSCC patients represents an important factor that predicts poor prognosis and resistance to chemo- and radiotherapy. The clinical application of survivin as a molecular target in HNSCC therapy significantly benefits HNSCC patients.

However, the present meta-analysis also had several limitations. First, we found that different paper qualities and sample sizes across the studies might cause bias in the meta-analysis. Second, the approaches used to evaluate the survivin expression were different, such as antibodies characters in IHC and instruments used in RT-PCR. Third, the cut-off value defining positive survivin expression varied among eligible studies (Table 1). Forth, our analysis might overestimate the prognostic significance of survivin to some degree due to the positive results reported in most of the including publications. Fifth, partial survival data of some including papers were extracted from Kaplan–Meier curves and may not as accurate as that obtained from original paper directly.

In conclusion, we searched the electronic databases and a total of 4891 patients in 28 studies were enrolled for meta-analysis, the results demonstrating that patients with survivin expression are more likely to have worse prognosis. Taken together, our meta-analysis results suggest that survivin gains a prognostic and diagnostic value for the HNSCC patients. However, more larger sample size studies are required to acquire a more representative and precise result.

## Supplementary Information

**Additional file 1: Table S1.** Characteristics of the antibodies used in IHC in the including studies.

## Data Availability

All data generated or analysed during this study are included in this published article [and its supplementary information files]. The data that support the findings of this study are available from the corresponding author upon reasonable request.
