# Supplementary material for: The prognostic significance of survivin expression in patients with HNSCC: a systematic review and meta-analysis
Source: BMC Cancer. 2021 Apr 17;21:424. doi: 10.1186/s12885-021-08170-3 (PMC8052826; doi:10.1186/s12885-021-08170-3)
Supplement: Supplementary file 1 — Additional file 1: Table S1. Characteristics of the antibodies used in IHC in the including studies. [file 12885_2021_8170_MOESM1_ESM.docx]

Table S1-Characteristics of the antibodies used in IHC in the including studies

| **Author** | **Clone** | **Dilution** | **Antigen Retrieval** | **Positive Signals** | **Source** |
| --- | --- | --- | --- | --- | --- |
| Elhadj[17] | Mouse monoclonae | 1:50 | Citric acid high pressure | Nuclear | Santa Cruz Biotechnology |
| Erpolat[18] | Rabbit polyclone | 1:50 | Citric acid high pressure | Nuclear, cytoplasmic | Neomarkers(Fremont, CA) |
| Fiedler[19] | Rabbit polyclone | 1:50 | Citric acid high pressure | Nuclear and in one case cytoplasmic | Abcam |
| Jin[20] | Mouse abtibody | 1:100 | Citric acid high pressure | Nuclear | Santa Cruz Biotechnology |
| Li[21] | Polyclonal antibody | 1:200 | Citric acid high pressure | Nuclear, cytoplasmic | Santa Cruz Biotechnology |
| Li[22] | Rabbit monoclone | 1:1000 | Citric acid high pressure | Nuclear, cytoplasmic | Cell Signaling Technology |
| Lin[23] | Rabbit polyclone | NR | Citric acid high pressure | Mainly cytoplasmic | Novus Biologicals(Littleton,USA) |
| Muzio [24] | Rabbit polyclone | 1:50 | Citric acid high pressure | NR | Novus Biologicals(Littleton,USA) |
| Preuss[25] | Rabbit polyclone | NR | Citric acid high pressure | Nuclear, cytoplasmic | Novus Biologicals(Littleton,USA) |
| Tastekin[26] | Rabbit polyclone | 1:100 | Citric acid high pressure | Nuclear, cytoplasmic | Novus Biologicals(Littleton,USA) |
| Wang[27] | Goat polyclone | 1:200 | Citric acid high pressure | cytoplasmic |  |
| Xiang[28] | Rabbit antibody | 1:100 | NR | Nuclear, cytoplasmic | Santa Cruz Biotechnology |
| Yip[29] | Rabbit polyclone | 1:50 | Citric acid high pressure | Nuclear, cytoplasmic | Novus Biologicals(Littleton,USA) |
| Zhao[30] | Polyclonal antibody | NR | Citric acid high pressure | Nuclear, cytoplasmic | Boster Biologicals,China |
| Dong [31] | Rabbit polyclone | 1:50 | Citric acid high pressure | Mainly cytoplasmic | Alpha Diagnosic International, USA |
| Zhang[33] | NR | NR | Citric acid high pressure | Cytoplasmic | Beijing Zhongshan Golden Bridge Biotechnology Co |
| Farnebo[34] | Rabbit polyclone | 1:400 | Citric acid high pressure | Nuclear, cytoplasmic | Thermo Fisher Scientific, UK |
| Freier[35] | Mouse monoclone | 1:3000 | Citric acid high pressure | Nuclear, cytoplasmic | Novus Biologicals(Littleton,USA) |
| Hansson[36] | Rabbit polyclone | 1:400 | Citric acid high pressure | Nuclear, cytoplasmic | Thermo Fisher Scientific, UK |
| Munscher[37] | Rabbit monoclone | 1:900 | Citric acid high pressure | Nuclear, cytoplasmic | Abcam |
| Pickhard[38] | Mouse monoclone | 1:20 | Citric acid high pressure | Nuclear | Dako Deutschland GmbH Hamburg, Germany |
| Su[39] | Rabbit polyclone | 1:500 | Citric acid high pressure | Mainly cytoplasmic | Santa Cruz Biotechnology |
| Troiano [40] | Rabbit polyclone | NR | Citric acid high pressure | Nuclear, cytoplasmic | Novus Biologicals(Littleton,USA) |
| Pizem[41] | Rabbit polyclone | 1:2000 | Citric acid high pressure | Nuclear, cytoplasmic | Novus Biologicals(Littleton,USA) |
| Marioni[42] | Mouse monoclone | 1:50 | Citric acid high pressure | Nuclear, cytoplasmic | Santa Cruz Biotechnology |
| Marioni[43] | Mouse monoclone | 1:51 | Citric acid high pressure | Nuclear, cytoplasmic | Santa Cruz Biotechnology |
| Kim[44] | Rabbit polyclone | 1:100 | Citric acid high pressure | Cytoplasmic | Abcam |
